# *In vitro* growth factor-induced bio engineering of mature articular cartilage

**DOI:** 10.1016/j.biomaterials.2012.09.076

**Published:** 2013-02

**Authors:** Ilyas M. Khan, Lewis Francis, Peter S. Theobald, Stefano Perni, Robert D. Young, Polina Prokopovich, R. Steven Conlan, Charles W. Archer

**Affiliations:** a Division of Pathophysiology and Repair, School of Biosciences, Cardiff University, Museum Avenue, Cardiff CF10 3AX, Wales, UK; b Centre of Nanohealth, Institute of Life Sciences, College of Medicine, Swansea University, Singleton Park, Swansea SA2 8PP, Wales, UK; c Institute of Medical Engineering & Medical Physics, School of Engineering, Cardiff University, The Parade, CF24 3AA, Wales, UK; d School of Chemical Engineering, University of Birmingham, Birmingham B15 2TT, UK; e School of Optometry and Vision Sciences, Cardiff University, Maindy Road, Cardiff University, Cardiff, CF24 4LU, Wales, UK; f School of Pharmacy and Pharmaceutical Sciences, Cardiff University, Cardiff CF10 3NB, Wales, UK

**Keywords:** Articular cartilage, Postnatal maturation, Biochemical, Repair

## Abstract

Articular cartilage maturation is the postnatal development process that adapts joint surfaces to their site-specific biomechanical demands. Maturation involves gross morphological changes that occur through a process of synchronised growth and resorption of cartilage and generally ends at sexual maturity. The inability to induce maturation in biomaterial constructs designed for cartilage repair has been cited as a major cause for their failure in producing persistent cell-based repair of joint lesions. The combination of growth factors FGF2 and TGFβ1 induces accelerated articular cartilage maturation *in vitro* such that many molecular and morphological characteristics of tissue maturation are observable. We hypothesised that experimental growth factor-induced maturation of immature cartilage would result in a biophysical and biochemical composition consistent with a mature phenotype. Using native immature and mature cartilage as reference, we observed that growth factor-treated immature cartilages displayed increased nano-compressive stiffness, decreased surface adhesion, decreased water content, increased collagen content and smoother surfaces, correlating with a convergence to the mature cartilage phenotype. Furthermore, increased gene expression of surface structural protein collagen type I in growth factor-treated explants compared to reference cartilages demonstrates that they are still in the dynamic phase of the postnatal developmental transition. These data provide a basis for understanding the regulation of postnatal maturation of articular cartilage and the application of growth factor-induced maturation *in vitro* and *in vivo* in order to repair and regenerate cartilage defects.

## 1 Introduction

Repair and regeneration of articular cartilage defects presents biologists and bioengineers with formidable challenges.

From the biological perspective, repair of adult cartilage is complicated in that this tissue is avascular, has a low cell to volume ratio and is rich in glycosaminoglycan containing proteoglycans producing a high negative fixed charge density [1]. These attributes limit active or passive cellular migration to the lesion site and characterise the poor intrinsic healing capacity of adult tissue [2]. Above a threshold defect diameter of 3–6 mm, cartilage lesions rarely heal spontaneously leading to progressive cartilage degeneration [3,4]. This latter impasse has been resolved to some extent through cell transplantation of culture expanded, autologous articular chondrocytes or mesenchymal stem cell populations into chondral defects [5-8]. Further complications arise as transplanted cells initially adopt an immature cartilage phenotype that appears to be subject to phenotypic instability [9], resulting in the inappropriate production of fibrocartilage or calcified tissue, both of which are to varying degrees deleterious to joint function [10,11].

From the viewpoint of a bioengineer, articular cartilage tissue engineering presents unique problems, namely the growth and maturation of implanted biomaterial constructs in an environment, the knee, where peak forces can be seven-times bodyweight [12] and where the joint can undergo an average of 5000 loading cycles during normal daily activities [13]. Whilst generation of neocartilage using autologous donor chondrocytes or stem cells seeded within fabricated scaffolds does frequently result in hyaline-like cartilage [14], there is no evidence to show that these tissues then form adult, mature cartilage with the bulk and interface properties consistent with the latter phenotype [15,16]. Hunziker (2009) has hypothesised the deficiencies of chondrocyte transplantation and matrix-assisted technologies in providing persistent cell-based repair of chondral lesions is due to an inability to induce the formation of functionally competent mature articular cartilage.

Neocartilage generated through tissue engineering has many similarities with foetal and immature articular cartilage. Immature cartilages are characterised by the relatively isotropic nature of collagen fibril structure and cellular organisation compared to adult cartilage. Mature cartilage has a pseudo-stratified structure composed of superficial, mid, deep and calcified zones [17,18] and the chondrocytes within each zone have specialised functions that homeostatically regulate bulk and/or interface properties [19,20]. In addition to a stratified structure, the arcade-like organisation of collagen fibrils and the subdivision of the extracellular matrix into pericellular, territorial and inter-territorial zones in mature cartilage are also critical factors in its ability to support physiological joint forces [21–23]. In terms of biochemical and biophysical properties mature cartilage is generally less elastic, smoother in appearance, has lower water content and higher collagen content with respect to wet weight than immature cartilage [1,24].

The developmental transformation of immature cartilage to one that is anisotropic in structure and adapted to site- and joint-specific function occurs postnatally in mammals and is stimulated by biochemical and biomechanical cues that, in the case of the former, some parts of which may be developmentally regulated [17,25]. Immature cartilage, therefore, serves as a transient template upon which the diversity of form and function may be elaborated [26]. The rate at which postnatal maturation of immature cartilage proceeds, to the point where there is no significant difference with mature cartilage varies from species to species, and is approximately two months, five months and 10–18 years postpartum for rabbit, horse and human cartilages, respectively [15,17,27]. The extended time of maturation and gradual adaptation of human joints to dynamic loading has clinical implications, in that intrinsic and extrinsic mechanisms for cartilage repair have to recapitulate this developmental transformation in order to generate durable repair tissue, especially in joints subjected to constant dynamic loading [15]. Therefore, understanding the mechanisms that regulate postnatal maturation is a critical step in advancing strategies for cell and biomaterial based therapies for cartilage repair. Insulin growth factor (IGF), transforming growth factor beta (TGFβ) and fibroblast growth factor (FGF) families of cytokines are known to regulate chondrocyte metabolism [28]. The levels of TGFβ1 and TGFβ2 rise postnally and that of IGF1 peaks at puberty indicating possible roles in tissue maturation [29,30]. Whilst IGF1 induces tissue growth and stimulation of proteoglycan deposition during *in vitro* culture of immature articular cartilage explants, it causes a reduction in tensile mechanical function, properties inconsistent with tissue maturation [31]. Developmental studies of TGFβ family member levels in articular cartilage indicate that while TGFβ1-3 are expressed prior to zonal stratification, following stratification TGFβ2 and TGFβ3 levels remain high whilst those of TGFβ1 are much lower [32]. However, studies of *in vitro* cultured immature cartilage explants show exogenously added TGFβ1 promotes tissue homeostasis with no overall change in their size, composition or biomechanical function [31]. FGF2 exerts opposing effects on immature cartilage, promoting mitosis and anabolism at low concentration (<30 ng/ml) but inhibiting the same processes at higher (30–300 ng/ml) concentration [33]. Combinations of factors such as FGF2 and TGFβ1 have synergistic effects on chondrocytes, repressing collagen type II gene expression [34], reducing the doubling time of culture expanded human chondrocytes and maintaining their phenotypic stability [35]. FGF2 and TGFβ1 have also been used in the tissue engineering context to dedifferentiate chondrocytes in order to induce cellular proliferation prior to using IGF1 to promote redifferentiation and growth [36].

Recent work has shown the combination of FGF2 (100 ng/ml) and TGFβ1 (10 ng/ml) induce precocious postnatal developmental maturation of immature articular cartilage *in vitro* [37]. The most conspicuous change within growth factor-treated cartilage explants is evidence of synchronised growth and resorption that results in thinner cartilage, mirroring morphological changes that occur during maturation *in vivo* [17,27]. Further evidence of maturation following growth factor treatment in cartilage explants are an increase in the ratio of mature to immature collagen crosslinking and developmentally encoded changes in extracellular distribution of proteins such as perlecan. We therefore hypothesised that key biophysical and biochemical properties characteristic of mature cartilage appear concomitantly with changes in morphology. This study used native immature and mature articular cartilage as references to determine if morphological transformation of growth factor-treated immature cartilage equated to biophysical and biochemical changes consistent with a transition to the mature phenotype.

## 2 Materials and methods

### 2.1 Materials

All chemicals were purchased from Sigma (Poole, UK) unless stated. Mature (over 18 month old) and immature (7-day-old) cartilage from bovine steers was obtained from local abattoirs. The age range of the mature donors was between 18 and 28 months.

### 2.2 Articular cartilage explant culture

Articular cartilage explants were surgically removed under sterile conditions from immature metacarpophalangeal joints. Full depth explants were excised using a 6 mm diameter biopsy punches (Stiefel Laboratories Inc, NC, USA) from the medial aspect of the medial condyle of individual joints. Explants were placed initially in Dulbecco's modified Eagles medium (DMEM; Invitrogen, Paisley, UK) and washed in the same medium to remove blood and small particulates due to the presence of a small amount of subchondral bone lining the basal aspect of cartilages. Explants were then cultured for 21 days in serum-free culture medium; DMEM (high glucose, 4.4 g ml^−1^), 100 μg ml^−1^ ascorbate-2-phosphate, 50 μg ml^−1^ gentamicin, 10 mm HEPES pH 7.5 (Invitrogen) supplemented with 10 μg ml^−1^ insulin, 5.5 μg ml^−1^ transferrin, 6.7 ng ml^−1^ selenium (ITS), with or without 100 ng ml^−1^ FGF2 and 10 ng ml^−1^ TGFb1 (Peprotech, London, UK). Explants cultures were placed in a humidified 5% CO_2_ incubator kept at 37 °C and culture medium was changed on every third day.

### 2.3 Imaging of articular cartilage

Eight micron sections of formalin-fixed cartilage samples that had previously been processed for wax embedding were hydrated and stained with 1% aqueous Safranin-O for 120 s and for 1 min in 1% haemotoxylin. For detection of birefringence by polarised light microscopy sections were hydrated and pretreated with 1% w/v bovine testicular hyaluronidase in Tris-buffered saline for 1 h at 37 °C then stained for 1 h in 0.1% Sirius red F3BA in saturated aqueous picric acid. Stained sections were briefly washed in 0.01N HCL, dehydrated, cleared and mounted in DPX mounting medium (RA Lamb, UK). For transmission electron microscopy, samples were prepared as previously described [37]. Sections were contrasted with uranyl acetate and lead citrate and examined with a JEOL 1010 transmission electron microscope equipped with a Gatan Orius SC1000 CCD camera.

### 2.4 Biochemical analyses

Explants were weighed wet, frozen, lyophilised, reweighed dry then assayed for sulphated glycosaminoglycan (sGAG) and hydroxyproline content. sGAG content was measured with the dimethylmethylene blue assay using explants that had been incubated for 120 min in papain digestion buffer, 20 mm sodium acetate pH 6.8, 1 mm EDTA, 2 mm dithiothreitol & 300 μg ml^−1^ papain at 60 °C [38]. Values of sGAG were determined against standards of shark derived chondroitin sulphate. Hydroxyproline content was determined by assaying acid hydrolysates of papain digested samples using the method of Creemers *et al*. (1997) [39].

### 2.5 AFM analysis

AFM experiments were carried out with a Nanowizard II (JPK Instruments, Berlin, Germany) equipped with nano-positioning sensors in all three axis and closed-loop feedback for precise, repeatable scanning and probe positioning with sub-nanometer resolution. AFM stiffness measurements were based on recording the elastic modulus of the cartilage material by using the AFM tip as a nanoindentor. Explants were washed in Hank's balanced salt solution for 5 min and then incubated in serum-free tissue culture media (DMEM). The Petri dish was then loaded onto a Nanowizard II Petri dish holder (JPK Instruments) platform and held at 37 °C while nanoindentation experiments were conducted. High aspect ratio etched silicon probes, dNTP10, (Bruker) of radius 20 nm with spring constants of 0.32 Nm^−1^ and resonant frequency 40–75 kHz were employed. Each cantilever used in the study was individually calibrated, calculating the sensitivity from a reference, hard force curve taken from the Petri dish surface. The cantilever-specific spring constants were calculated using the inbuilt thermal noise method of the Nanowizard instrument. A maximum load force of 20 nN was found to be optimal and applied to the surface in each recorded force curve. The cantilever approach and retraction velocity was constant, set at 1.8 μm s^−1^. The Poisson ratio was assumed to equal 0.5. Nanoindentation force experiments were conducted capturing 100 indentation curves in each scan area (10 × 10 μm) of the explant surface. These data represented the basis for the estimation of a sample's adhesive properties and Young's modulus (E) using Hertzian mechanics [40,41]. Manipulation of both the approach and retraction curves yields different measurements which were related to cell micromechanical properties and adhesion respectively. The material stiffness is the slope of the unloading curve at the maximum penetration depth, and, the minimum of the retraction curve is the force needed to overcome the adhesion between the sample and the probe. The Hertz model describes the simple case of elastic deformation of two perfectly homogeneous smooth bodies touching under load. The Hertz model presumes the indented sample is extremely thick in comparison to the indentation depth. This was the case here, where the indentation depth at a trigger force of 20 nN was always <10% of the cartilage thickness and thus below Bueckle's indentation depth limit [42].

### 2.6 Surface topography analysis

AFM was also employed to analysis the surface topography of cartilage samples. Images of the surfaces were recorded using tapping mode with silicon tips. Coordinates of the surfaces were acquired on a scan area of 25 × 25 μm and 256 × 256 points. Surface features such as asperity heights and their curvature radius were calculated according to Prokopovich and Perni (2010) [43]. In brief, asperities are located as points on the surface whose coordinates are higher than those surrounding. The 2D profile of the asperity is fitted with a parabola and the curvature radius of the asperity tip is calculated from the parabolic equation parameters. The height and curvature radius of each asperity is determined and the distribution determined at the end.

### 2.7 Friction testing

The frictional coefficient of cartilage explants was assessed using a pin-on-plate tribometer, with phosphate-buffered saline (PBS) as a lubricant. Six millimeter diameter freshly isolated and cultured articular cartilage explants, were fixed onto a nylon housing using cyanoacrylate. PBS was applied evenly over the polished glass surface, providing an average depth of approximately 1 mm. The tissue was then preloaded at 0.1 MPa for 120 s prior to disc rotation to ensure consistent boundary lubrication as described previously with Neu *et al* [44]. The sliding speed was then ramped to 12 mm/s, before data was recorded for 15 s. Retrospective analysis to compute the mean frictional coefficient was then completed using MS Excel (Microsoft, Redmond, WA, USA).

### 2.8 Immunofluoresence analysis of cartilage

Cultured or freshly isolated articular cartilage explants were frozen in n-hexane cooled in a bath of dry ice and ethanol. Tissues were then mounted in optimal cutting temperature embedding medium (Thermo Scientific, Epsom, UK) and eight micron sections cut using a Bright OTF500 cryostat (Bright Instrument Co. Ltd, Huntingdon, UK). Sections were dried and stored in foil at −20 °C until use. Tissue sections were ringed with wax pen, washed in Tris-buffered saline including 0.1% Tween-20 (TBS/T) and then blocked in 10% goat serum for 30 min. Primary monoclonal antibody anti-human collagen type I (Sigma–Aldrich, Poole, UK) was diluted 1:1000 in TBS/T then placed on sections and incubated overnight at 4 °C. Sections were washed three times in TBS/T then incubated with a 1:100 dilution of goat anti-mouse FITC conjugated secondary antibodies for 1 h at room temperature. Sections were washed repeatedly in TBS/T then coverslipped following the application of an anti-bleaching reagent, Vectashield (Vectorlabs, Peterborough, UK), containing propidium iodide as a nuclear marker.

### 2.9 Quantitative reverse transcription-polymerase chain reaction (qRT-PCR)

Cultured or freshly isolated articular cartilage explants were frozen in n-hexane as described above and stored at −70 °C prior to RNA extraction. Frozen explants were homogenised in the presence of frozen 0.5 ml TRI reagent using a mikro-dismembrator U and chilled steel vessels (B. Braun Biotech International, Melsungen, Germany). The supernantant from the latter process was placed in an RNAEasy column for total RNA extraction with DNAseI digestion step (Qiagen, Crawley, UK). Total RNA was quantified using NanoDrop 2000 spectrophotometer (NanoDrop, Wilmington, USA). One microgram of total RNA was used for reverse transcription reaction using the GoScript kit utilizing M-MLV reverse transcriptase and random primers (Promega, Southampton, UK). QPCR reactions were performed using the GoTaq qPCR mastermix (Promega), 12.5 ng cDNA and 0.3 mm forward and reverse primers. Reactions were performed on a Stratagene Mx3000 real-time PCR analyser (Agilent Technologies, Edinburgh, UK) with the following thermal cycling program; 95 °C for 10 min^−1^ cycle, 95 °C 30 s, 55 °C 60 s, 72 °C 30 s–40 cycles. Standard curves over the linear range of amplification were generated for all primer sets, and data was used where the efficiency of amplification was between 90% and 105% and the melt curves generated a single product. The data shown is the ratio of the concentration of the gene of interest (in nanogrammes) and 18S rRNA (in nanograms). The nucleotide sequences of primer sets used in this study were as follows; collagen type IαI forward 5′ TAC GCCCCA CCA GTC ACC TGC GTA C 3′ reverse 5′ GTT TCC ACA CGT CTC GGT CA 3′, RSP18F 5′ CAC TGG AGG CCT ACA CGC CG 3′ and RSP18B 5′ AGG CAA TTT TCC GCC GCC CA 3′.

### 2.10 Statistical analysis

PASW18 (IBM, NY, USA) was used to statistically analyse these data. All datasets were checked for normal distribution using the Shapiro–Wilk test and homogeneity of variances using Levene's test prior to statistical analysis. For parametric analysis of 2 groups we used two-tailed Student's paired sample *t*-test, and for multiple groups a one-way analysis of variance test (ANOVA). The Bonferroni correction factor was applied to avoid false positive results due to multiple testing. Where data was either not normally distributed or variances not equal non-parametric Kruksal-Wallis and Mann–Whitney *U* tests were used for multiple and paired groups, respectively. The sample size, N, of each study represents explants excised from individual donor animals.

## 3 Results

### 3.1 Growth factor-induced postnal maturation of cartilage

**Fig. 1:**
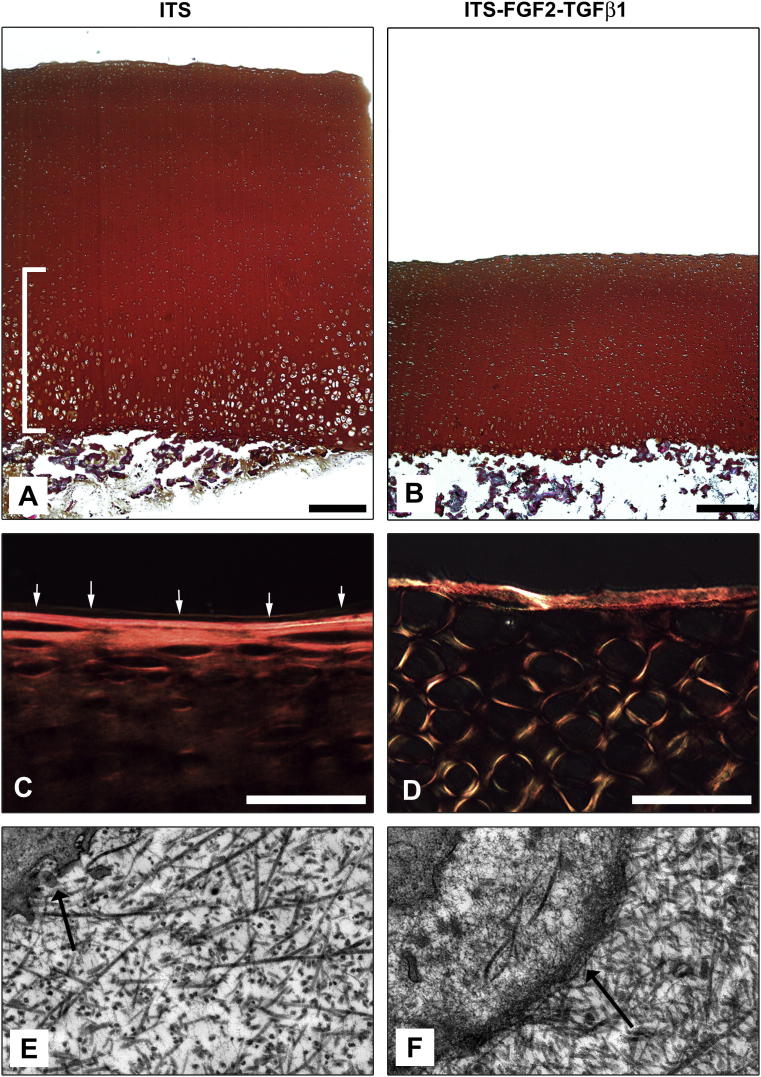
**FGF2 and TGFβ1 induce potent morphological changes in immature articular cartilage during *in vitro* culture**. Explants were taken from adjacent sites from the same joint and cultured either in the absence (A) or continual presence (B) of 100 ng ml^−1^ FGF2 and 10 ng ml^−1^ TGFβ1 for 21 days in ITS containing serum-free medium. Growth factor-treated explants undergo significant resorption resulting in the disappearance of hypertrophic chondrocytes that reside in the deep zone of immature articular cartilage (bracketed in (A)]. Bar equals 500 μm. Polarising light microscopy of picro-sirius red stained sections of untreated (C) and growth factor-treated cartilage explants (D). The surface cartilage of growth factor treated cartilage displays extensive changes in collagen orientation many fibrils are anti-parallel to the surface axis (D). Also, a thin fluorescent line parallel to the surface (white arrows) delineates the lamina splendens, a collagen and lipid rich structure that is approximately 3 microns deep. This structure is absent in growth factor-treated cartilage. Bar equals 50 μm. Electron microscopy of surface chondrocytes (×7500) in untreated (E) and growth factor-treated (F) cartilage explants. Note the appearance of a thickened pericellular coat surrounding individual surface chondrocytes in growth factor-treated cartilage (black arrow in F) compared to untreated cartilage where this micro-anatomical unit of mature chondrocytes is absent (arrow in E). Also noteworthy is the increased collagen fibril density in growth factor-treated cartilage explants (F). (For interpretation of the references to colour in this figure legend, the reader is referred to the web version of this article.)

*In vitro* culture of articular cartilage explants excised from immature metacarpophalangeal joints in the presence of FGF2 (100 ng ml^−1^) and TGFβ1 (10 ng ml^−1^) for 21 days results in profound morphological change [37]. This change is illustrated by tissue resorption from the deep zone resulting in a reduction in height of approximately 50% of growth factor-treated explants when compared to paired control explants excised from directly adjacent sites of the same joint and cultured in serum-free medium alone (272.5 ± 37.8 μm vs 143.5 ± 33.7 μm, respectively, *P* < 0.001, *N* = 4) Fig. 1A–B. When viewed under polarising conditions by light microscopy, we observed changes in the pattern of birefringence surrounding individual chondrocytes in the surface zone of growth factor-treated explants when compared to control untreated explants where the birefringence signal was weaker and parallel to the immediate surface, Fig. 1C–D. An increase in birefringence occurs as a result of enhanced alignment or bundling of collagen fibrils [45]. High resolution imaging of surface chondrocytes using transmission electron microscopy revealed that surface zone chondrocytes in growth factor-treated cartilage, corresponding to those cells displaying high birefringence, possessed a pericellular envelope enclosing a proteoglycan rich glycocalyx. In comparison, surface chondrocytes of explants cultured in control, serum-free medium were not separated from the territorial matrix by a pericellular coat, Fig. 1D–E.

### 3.2 Biochemical analyses of *in vitro* maturation

Biochemical analyses of native and cultured articular cartilage explants demonstrated that growth factor-treated cartilage was significantly different to both immature and serum-free cultured immature cartilage explants in water content (*P* < *0.05*, *N* = 6), dry/wet weight ratio (*P* < *0.05*, *N* = 6) and hydroxyproline content (*P* < *0.05*, *N* = 6), Table 1. There was no statistical difference in the latter values when growth factor-treated cartilage was compared to native mature cartilage. There was no statistical difference in sGAG values within native or cultured explant sample groups.

### 3.3 AFM analyses of cartilage stiffness and adhesiveness

**Fig. 2:**
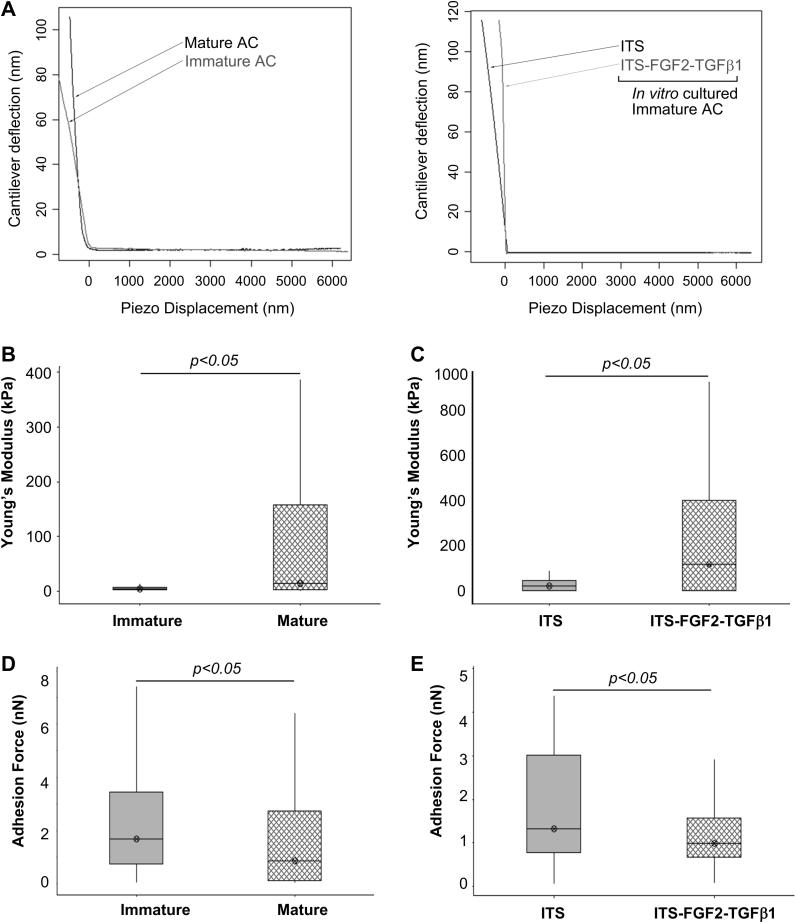
**Changes in nanoscale elasticity and adhesive properties at the apical surface of articular cartilage**. Representative load–displacement curves for nanoindendation analysis of cartilage samples (A). Despite having heterogeneous surfaces [48,52], freshly isolated immature cartilage explants exhibited significantly different ranges of both elasticity (B) and adhesion (D) when compared to their mature tissue sample counterparts. These differences are visualised in the boxplot representation and were shown to be significant through Mann–Whitney statistical analysis; *P* < 0.05. Similar results were observed for elasticity and adhesion measurements for immature growth factor-treated cartilage explants and their untreated controls (C, E). A significant (*P* < 0.05) increase in sample Young's modulus, and therefore, stiffening of the surface was observed following growth factor treatment when compared to the untreated control samples (C). A decrease in the adhesive status of the surface of growth factor-treated cartilage was observed compared to the control samples, a significant lowering of the 50% interquartile range is depicted in the boxplot analysis, indicating a reduction in the maximum force needed to withdraw the AFM stylus from the sample surface (E). This behavioural characteristic was reversed when analysing the approach curves and sample elasticity of cartilages where we observed an increase in the 50% interquartile range (B, C).

One key property of maturing cartilage is a progressive increase in material stiffness [46]. Measuring the nanoscale compressive strength of cartilage using AFM we observed freshly isolated mature articular cartilage showed an increase in the median values for Youngs's modulus (*E*_*ind*_), and therefore a strengthening or hardening of the apical surface compared to immature cartilage (13.28 kPa interquartile range 2.81–35.34 versus 4.658 kPa interquartile range 3.199–7.582, respectively, *P* < *0.02*, *N* = 6), Fig. 2A–B. We also observed an increase in median nano-compressive stiffness between immature cartilage explants that had been cultured for 21 days with growth factors FGF2 and TGFβ1 compared to explants cultured in serum-free medium (97.8 kPa interquartile range 4.82–244.4 versus 25.22 kPa interquartile range 3.41–49.60, respectively, *P* < *0.02*, *N* = 6). The elastic moduli values obtained for the treated samples were more variable than the untreated cartilage and that of the mature cartilage controls. Freshly isolated immature cartilage also exhibited significantly higher maximal adhesion forces than mature cartilage (1.67 nN interquartile range 0.74–3.43 versus 0.84 nN interquartile range 0.10–2.72, *P* < 0.02, *N* = 6, respectively) and this trend was also replicated when maximal adhesive forces of unstimulated immature cartilage explants were compared to growth factor-treated immature cartilage (1.32 nN interquartile range versus 0.98 nN interquartile range 0.68–1.58, *P* < *0.02*, *N* = 6, respectively), Fig. 2C.

### 3.4 Surface topography

**Fig. 3:**
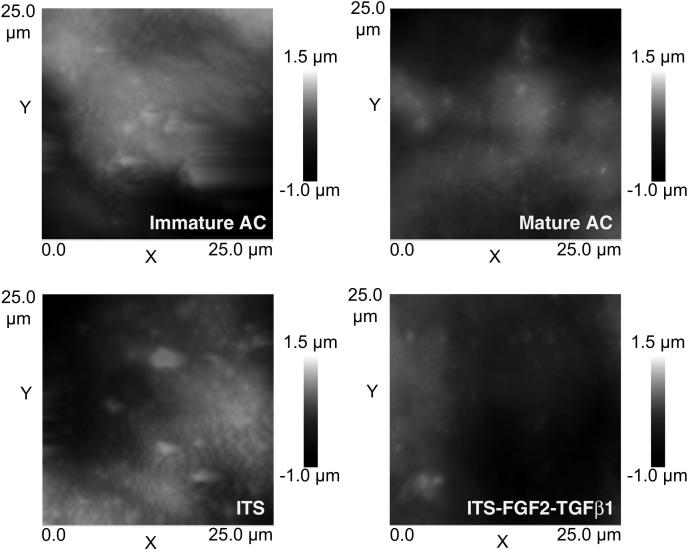
**AFM surface scan of freshly isolated immature and mature, and, serum-free (ITS) and growth factor-treated (ITS-FGF2-TGFβ1) cultured articular cartilages.** Roughness analysis (*see*Table 2) revealed that mature cartilages and samples treated with growth factors were smoother than immature cartilages or control samples. In addition, we also note that in surface scans, mature and growth factor-treated (ITS-FGF2-TGFβ1) cartilage appeared more fibrous and exhibit a less dimpled appearance than immature or cultured immature (ITS) cartilages.

Examples of the surface topography examined using AFM are shown in Fig. 3. It is evident that native mature cartilage is smoother than immature cartilage, similarly samples treated with growth factors appear smoother than control. Quantitative assessment of the surface roughness through the values of Root Mean Square (R_RMS_), defined as the standard deviation of the asperity heights, is presented in Table 2. These results confirm that following *in vivo* maturation cartilage become smoother (*P* < *0.05*, *N* = 6) as R_RMS_ decreases; also the addition of growth factors results in a lower values of surface roughness than the control samples (*P* < *0.05*, *N* = 6). Furthermore, there was no difference in R_RMS_ values between mature cartilage and growth factor-treated cartilage explants (*P* > *0.05*, *N* = 6), indicating as previously demonstrated for biochemical data a convergence in biophysical properties.

Each asperity, beside its height, is defined by the curvature radius of the extremity. The distributions of such curvature radii, for each of the samples, did not follow a Gaussian profile (*data not shown*); for this reason percentile values are presented instead of mean and standard deviation in Table 2. Curvature radii of native or growth factor-induced mature cartilages are greater than those of control or native immature cartilages.

### 3.5 Friction analysis of cartilage

**Fig. 4:**
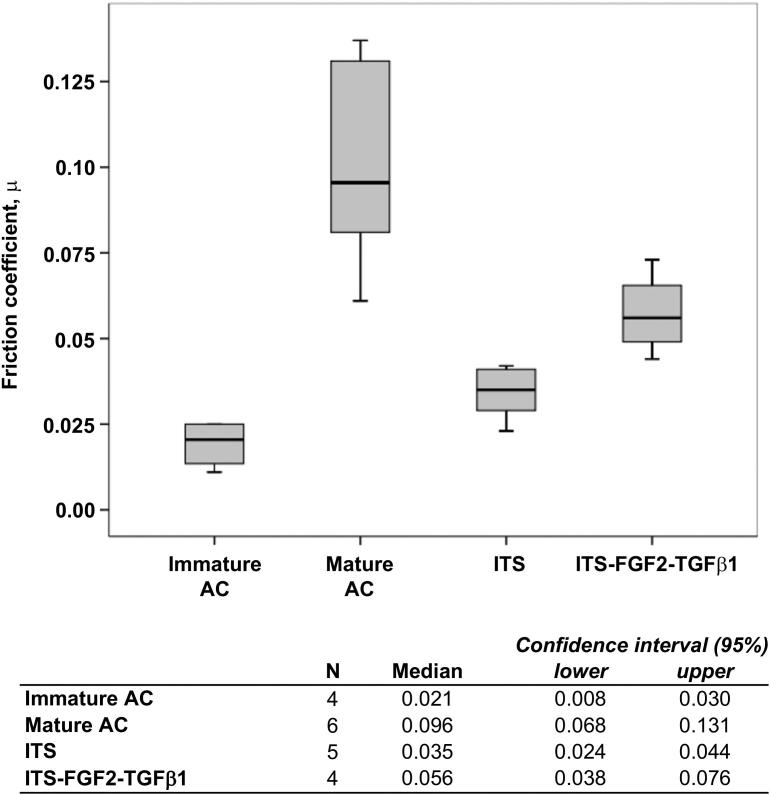
**Biotribological analysis of articular cartilage explants**. The coefficient of friction (CoF) was measured for freshly isolated immature (7-day-old) and mature (>18 month old) cartilages, and, *in vitro* cultured growth factor-treated and untreated immature cartilages (*see Materials and Methods*). The CoF of freshly isolated mature cartilages was significantly higher than their immature counterparts (*P* < 0.01). The CoF of growth factor-treated explants (ITS-FGF2-TGFβ1) was also significantly higher than untreated (ITS) explants (*P* < 0.01). The CoF of growth factor-treated cartilage explants increased approximately 3-fold (*P* < 0.05) following *in vitro* culture for 21 days compared to freshly isolated immature tissue.

The mean equilibrium frictional coefficient of freshly isolated mature cartilage was significantly higher (4.6-fold) than its immature counterpart (*P* < 0.01, *N* = 4), Fig. 4. Similarly, the frictional coefficient of growth factor stimulated cartilage explants were also 1.6-fold higher than explants cultured in medium lacking growth factor (*P* < *0.014*, *N* = 5). Experimentally induced *in vitro* maturation caused an approximately 3-fold rise in friction coefficient (*P* < 0.02, *N* = 4) compared to freshly isolated immature cartilage.

### 3.6 Collagen gene expression in cartilage

**Fig. 5:**
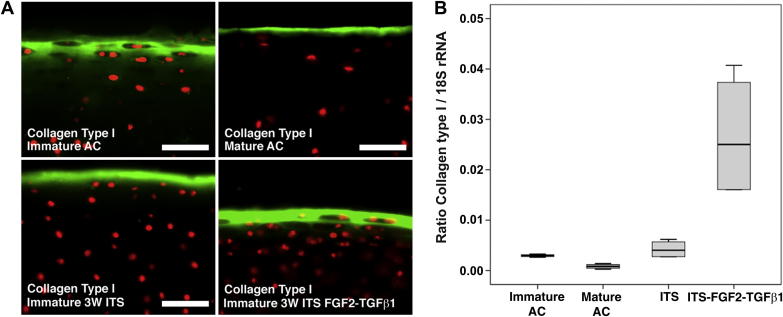
**Collagen type I expression during *in vivo* and *in vitro* articular cartilage developmental maturation**. In freshly isolated tissue, labelling for anti-collagen type I antibodies was observed as a broad and diffuse surface layer, and, localised as a thinner layer at the surface in mature cartilage (A). Labelling in cartilage explants cultured in serum-free (ITS) or growth factor medium (ITS-FGF2-TGFβ1) was more intense and was consolidated at the surface of these cartilages. Bar equals 50 μm. The ratio of collagen type IαI (in nanograms) normalised to 18S rRNA (in nanograms) is shown (B). Transcript levels of collagen type IαI decrease approximately 3-fold (*P* < 0.05) as cartilage matures, but following *in vitro* experimental maturation transcript levels increase approximately 6-fold compared to serum-free cultured (ITS) cartilage explants (*P* < 0.02).

Having observed changes in the biomechanical and frictional properties at the apical interface of cartilages examined in this study we investigated whether these differences correlated with the presence of the surface structural protein, collagen type I. Using fluorescence microscopy we observed a contraction of, and, weaker antibody labelling for collagen type I in the progression from native immature to mature cartilage, Fig. 5A. A relatively thinner, consolidated band of labelling was also evident in *in vitro* cultured control cartilage explants compared to native immature cartilage, and this band was noticeably more intense in growth factor stimulated immature cartilages Fig. 5A. Using qPCR analysis, we observed that collagen type Iα1 gene expression decreased 3.5-fold in native mature cartilage compared to its immature counterpart (*P* < *0.05*, *N* = 4). However, collagen type Iα1 expression levels rose 6.3-fold in growth factor stimulated cartilage explants compared to cartilage explants cultured in control serum-free medium (*P* < *0.02*, *N* = 4), Fig. 5B, indicating active remodelling of the surface zone.

## 4 Discussion

One of the major impediments in designing and implementing cartilage repair procedures using cellular and biomaterial composites has been the inability to induce and/or accelerate maturation *in vitro* or *in vivo*. Immature articular cartilage is the template upon which biomechanical and biochemical cues act in adapting the tissue to joint-specific function through induction of morphological, structural and biomolecular heterogeneity [26]. Data from equine studies, has shown that structural and biochemical heterogeneity of joint cartilage is delayed upon exercise deprivation in immature animals [47]. In the latter study, Brama *et al*. (2002) also demonstrated that access to exercise after deprivation significantly delayed the appearance of tissue heterogeneity, and, in some collagen-related parameters was incomplete. Therefore, articular cartilage maturation is partly a developmentally encoded process, and is not recapitulated fully in more mature animals through biomechanical conditioning alone. These latter studies are of great significance because the cellular components of biomaterials composites are principally embryonic or adult mesenchymal stem cells that produce immature neocartilage when induced to differentiate. They also provide a rational basis to explain why, in part, matrix-assisted repair strategies frequently do not lead to healing of cartilage lesions and what is required to overcome such obstacles.

Previous work has shown that *in vitro* culture of immature articular cartilage in the presence of FGF2 and TGFβ1 growth factors induces morphological changes that are consistent with the postnatal developmental transition to tissue maturity [17,37]. These changes included a reduction in cartilage height of 50% through resorption of the deep/epiphyseal-derived zone directed by co-ordinated expression of matrix metalloproteinases −1, −13, −2 and −9, and, tissue inhibitor of metalloproteinases 1–3 [37]. The decrease in height and the loss of hypertrophic chondrocytes correlates with observations in studies of postnatal maturation in the patellofemoral joints of mice and rabbits [17,48]. Resorption in the deep zone of growth factor-treated explants is balanced by growth from the surface zone driven by chondroprogenitor stem cells. Our current data provides quantitative evidence that *in vitro* growth factor-induced differentiation of immature articular cartilage results in biophysical and biochemical properties that are indistinguishable from native mature cartilage.

The mean percentage water content and dry to wet weight ratio of growth factor-treated cartilage explants were identical to values obtained for native mature cartilages. As articular cartilage matures there is a progressive decrease in water content [49]. With extended culture *in vitro*, in free swelling conditions, the water content of immature cartilage increases, however growth factor-treated explants not only resisted accumulation of water during culture but the water content values converged to those found in native mature explants and were significantly less than mean value for native immature cartilage. [46]. Also, as articular cartilage maturation proceeds, the dry to wet weight ratio of tissue increases. [49]. We observed that growth factor-treated immature cartilage had identical dry to wet weight ratios to mature cartilage, correlating with a significant increase in hydroxyproline (collagen) content in this experimental group. There was no difference in sGAG (proteoglycan) content between native immature and mature, or, control and growth factor-treated cartilages and this pattern of data is similar to that described by Williamson *et al*. (2003) who observed no significant difference in the sGAG content between calf and young adult articular cartilage from the patellar-femoral groove [49].

In the latter study (Williamson *et al*., 2003), measurements of dynamic modulus showed a positive correlation (*r*^2^ = 0.42, *P* < *0.01*) with collagen content. We used AFM to measure the nanoscale biomechanical properties of cartilages, where we observed that both growth factor-treated and mature cartilages were significantly less elastic than control and immature cartilages, respectively. For this study we used nanometre-sized AFM tips that have been shown previously by Stolz *et al*. (2009) to distinguish differences in nano-compressive stiffness of articular cartilage from femoral heads as a function of aging [48]. We observed that the experimental stiffness values for cultured explants were significantly higher than those for freshly obtained tissue. The latter increase was due to a reduction in glycosaminoglycan content in *in vitro* cultured explants compared to freshly isolated native cartilages that had the general effect of raising the observed stiffness of these samples, also noted by Stolz *et al*. (2009) as a phenomenon occurring in human cartilage during aging. There was no significant difference in sGAG values between native, or, cultured explants, therefore negating proteoglycan content as a factor for increases in nano-compressive stiffness within the experimental group. The nanoscale data is determined by the sharpness and shape of the cantilevered probe. The conical probes used in this study were 20 nm in diameter and therefore are interrogating the surface at the same scale as proteoglycans and collagen fibrils, which form the structural components of the extracellular matrix. It is hypothesised that differences in surface stiffness during cartilage maturation measured at the nanometer scale represent changes in collagen structure, particularly in the thickness or density of fibres that can be modulated by fibril associated proteoglycans such as biglycan or decorin [50–52].

We also observed a similar pattern of results in nanoscale adhesion properties, where growth factor-treated and native mature cartilages exhibited reduced adhesion compared to untreated and native immature cartilages. These data led us to investigate the surface topography of cartilages, where we discovered that surface roughness declines approximately 40% between freshly obtained immature and mature cartilage, and 31% between control and growth factor-treated cartilage. The R_RMS_ values for native mature and growth factor-treated immature cartilage were not significantly different again indicating a convergence of biophysical properties to ones that characterise mature articular cartilage. Furthermore, the radii of asperities increased in mature and growth factor-treated cartilages by approximately the same magnitude. These data suggest as cartilage matures, the surface becomes stiffer and exhibits smoothing that results in a reduction of surface adhesion properties. These results confirm the findings of Ghadially *et al*. (1978) who observed by scanning electron microscopy ‘innumerable humps’ in immature articular cartilage from feline femoral condyles that in more mature cartilage were reduced in number and height, thus, revealing a comparatively smoother but more wrinkled surface [53]. Hump-like features were also present in native and cultured immature cartilages, but their appearance was reduced in native mature and *in vitro* matured cartilage.

The equilibrium frictional coefficient of native mature articular cartilage was greater than that of corresponding immature tissue, and, growth factor-treated cartilage greater than native and cultured control immature cartilages. The magnitude of friction is potentially influenced by factors including the surface roughness, stiffness and fluid pressurisation (liable to vary with cartilage thickness). The reduction in asperity height (i.e. roughness) would, in isolation, be expected to correlate with mature cartilage demonstrating a decreased frictional coefficient. That the frictional coefficient increases during growth factor-induced maturation suggests that a concomitant increase in stiffness reduces the tissue deformation to such an extent that a greater (frictional) force is required to slide one surface over the other. This reasoning may explain, in part, why loss of PRG4 (lubricin) expression in a mouse knockout model leads to precocious wear only in mature joints [51]. The cartilage thickness could have also influenced the lubrication and thereby the extent of friction, through the generation of differential fluid pressures (i.e. a greater thickness has the potential to generate higher fluid pressure, thereby enhanced lubrication and a lower frictional coefficient). Whilst the relatively thick (mature and growth factor-treated) cartilages do indeed exhibit lower frictional coefficients, the potential influence of variable fluid pressurisation was controlled by the application of a 0.1 MPa pre-load over a 120 s period, to enforce boundary lubrication conditions as per Neu *et al*. (2010) [44].

Collagen type I is expressed by surface chondrocytes in foetal and neonatal articular cartilage. As joints mature, gene expression and protein levels of collagen type I decline and it is progressively replaced by collagen type II [54]. In the metacarpophangeal joint of bovine steers we observed a contraction in the depth of antibody labelling for collagen type I from the surface, correlating with maturation of the joint surface from calf to young adult. This contraction of labelling was not observed in growth factor-treated cartilage explants. When collagen type I gene expression was quantitatively assayed, whilst there was as predicted a decrease in expression in native cartilage in the transition to maturity, we observed an approximate 6-fold increase in gene synthesis compared to control untreated cartilage. The function of collagen type I in surface articular cartilage during postnatal development has not been defined; the presence of collagen type I fibrils on the surface cartilage may confer greater resistance to shear stresses and/or may act as a template for, or initiator of, collagen type II quaternary structure formation. In this particular instance, increased collagen type I expression is a biomarker symbolising that growth factor-treated articular cartilage is still within a dynamic phase of maturation.

Data from studies examining neo-natal joint development have noted that anisotropy – a fundamental marker of tissue maturation – is initiated from the surface zone of articular cartilage [55,56]. We have also noted anisotropy of collagen fibril orientation in surface chondrocytes of growth factor-treated explants. However, the classical ‘arcade-like’ orientation of collagen fibrils is not present in growth factor-treated cartilages. Our studies were conducted in free swelling condition and in the absence of dynamic loading, and therefore, even though the cardinal biochemical and biophysical properties indicate attainment of the mature cartilage state, clearly this process in incomplete. Biochemical and biophysical heterogeneity are induced *in vivo* through adaption to dynamic loading, and this force is probably required ultimately to condition articular cartilage, and orientate collagen fibrils at the macroscale, so that it can attain equilibrium with its immediate environment.

## 5 Conclusion

We have quantitatively demonstrated that *in vitro* growth factor-induced maturation of immature articular cartilage causes the appearance of biomechanical and biophysical properties characteristic of mature adult cartilage. This process is rapid, dynamic and begins at the surface zone of articular cartilage. It is acknowledged that many cartilage repair procedures using cell and biomaterial constructs produce cartilage that is immature in phenotype and therefore deficient in many respects to restore normal, long-lasting, joint function. We hypothesise that the combined application of FGF2 and TGFβ1 to induce maturation of chondrocytes and/or synthetic constructs, *in vitro* or *in vivo*, should enable this fundamental stumbling block in cartilage repair to be overcome.

## Figures and Tables

**Table 1:** Comparison of biochemical composition measurements of freshly isolated immature and mature cartilage, and, *in vitro* cultured (ITS) and growth factor-treated (ITS-FGF2-TGFβ1) immature articular cartilages.

	Immature AC	Mature AC	Immature AC	
			ITS	ITS-FGF2-TGFβ1
% water (mg)/wet weight (mg)	73.8 ± 1.07	68.7 ± 1.67^a^	77.7 ± 1.70	68.7 ± 2.54^b^^a^
dry/wet weight ratio	0.26 ± 0.01	0.31 ± 0.02^a^	0.22 ± 0.02	0.31 ± 0.01^b^^a^
hydroxyporoline μg ml^−1^	6.65 ± 1.02	9.83 ± 2.07^nsd^	5.63 ± 0.93	8.30 ± 0.88^b^
sGAG μg ml^−1^	54.2 ± 3.87	49.6 ± 9.36^nsd^	38.2 ± 7.96	42.7 ± 6.79^nsd^

nsd, no significant difference to control groups. (*N* = 6 for all groups, *P* < 0.05).

a Significantly different from Immature AC.

b Significantly different from serum-free cultured (ITS) immature cartilage.

**Table 2:** Surface roughness analysis using AFM of freshly isolated immature and mature, and, immature cartilage explants cultured either in serum-free medium (ITS) or with growth factors (ITS-FGF2-TGFβ1).

	Immature AC	Mature AC	Immature AC	
			ITS	ITS-FGF2-TGFβ1
RMS (μm)	0.83 ± 0.15	0.49 ± 0.10	0.79 ± 0.12	0.55 ± 0.09^a^^,^^b^
25th percentile asperity radii (μm)	2.04	3.12	2.29	3.02
50th percentile asperity radii (μm)	3.04	4.58	2.72	4.62
75th percentile asperity radii (μm)	4.90	9.03	4.27	8.77

(*N* = 6 for all groups, *P* < 0.05)

a Significantly different from serum-free cultured (ITS) immature cartilage.

b Significantly different from Immature AC.
